# Proteome Mapping of a Cyanobacterium Reveals Distinct Compartment Organization and Cell-Dispersed Metabolism^1^

**DOI:** 10.1104/pp.19.00897

**Published:** 2019-10-02

**Authors:** Laura L. Baers, Lisa M. Breckels, Lauren A. Mills, Laurent Gatto, Michael J. Deery, Tim J. Stevens, Christopher J. Howe, Kathryn S. Lilley, David J. Lea-Smith

**Affiliations:** a Department of Biochemistry, University of Cambridge, Cambridge CB2 1QW, United Kingdom; b Computational Proteomics Unit, Cambridge Centre for Proteomics, University of Cambridge, Cambridge CB2 1QW, United Kingdom; c School of Biological Sciences, University of East Anglia, Norwich Research Park, Norwich NR4 7TJ, United Kingdom; d MRC Laboratory of Molecular Biology, Cambridge CB2 0QH United Kingdom

## Abstract

Cyanobacteria are complex prokaryotes, incorporating a Gram-negative cell wall and internal thylakoid membranes (TMs). However, localization of proteins within cyanobacterial cells is poorly understood. Using subcellular fractionation and quantitative proteomics, we produced an extensive subcellular proteome map of an entire cyanobacterial cell, identifying ∼67% of proteins in *Synechocystis* sp. PCC 6803, ∼1000 more than previous studies. Assigned to six specific subcellular regions were 1,712 proteins. Proteins involved in energy conversion localized to TMs. The majority of transporters, with the exception of a TM-localized copper importer, resided in the plasma membrane (PM). Most metabolic enzymes were soluble, although numerous pathways terminated in the TM (notably those involved in peptidoglycan monomer, NADP^+^, heme, lipid, and carotenoid biosynthesis) or PM (specifically, those catalyzing lipopolysaccharide, molybdopterin, FAD, and phylloquinol biosynthesis). We also identified the proteins involved in the TM and PM electron transport chains. The majority of ribosomal proteins and enzymes synthesizing the storage compound polyhydroxybuyrate formed distinct clusters within the data, suggesting similar subcellular distributions to one another, as expected for proteins operating within multicomponent structures. Moreover, heterogeneity within membrane regions was observed, indicating further cellular complexity. Cyanobacterial TM protein localization was conserved in Arabidopsis (*Arabidopsis thaliana*) chloroplasts, suggesting similar proteome organization in more developed photosynthetic organisms. Successful application of this technique in *Synechocystis* suggests it could be applied to mapping the proteomes of other cyanobacteria and single-celled organisms. The organization of the cyanobacterial cell revealed here substantially aids our understanding of these environmentally and biotechnologically important organisms.

## Abstract (precis)

The most extensive proteome map of an entire cyanobacterial cell demonstrates that thylakoid and plasma membrane proteins have distinct functions and that metabolic pathways are dispersed throughout the cell.

Cyanobacteria (oxygenic photosynthetic bacteria) are a widespread and abundant phylum of environmental and biotechnological importance (Zwirglmaier et al., 2008; Ducat et al., 2011). Among prokaryotes they are distinguished by the presence of a highly differentiated series of internal thylakoid membranes (TM), parts of which are in close contact, but do not fuse with the plasma membrane (PM; Rast et al., 2019). The cell envelope is similar to other Gram-negative bacteria, consisting of the PM, peptidoglycan layer, and outer membrane (OM; Stanier and Cohen-Bazire, 1977; Fig. 1).

**Figure 1.**
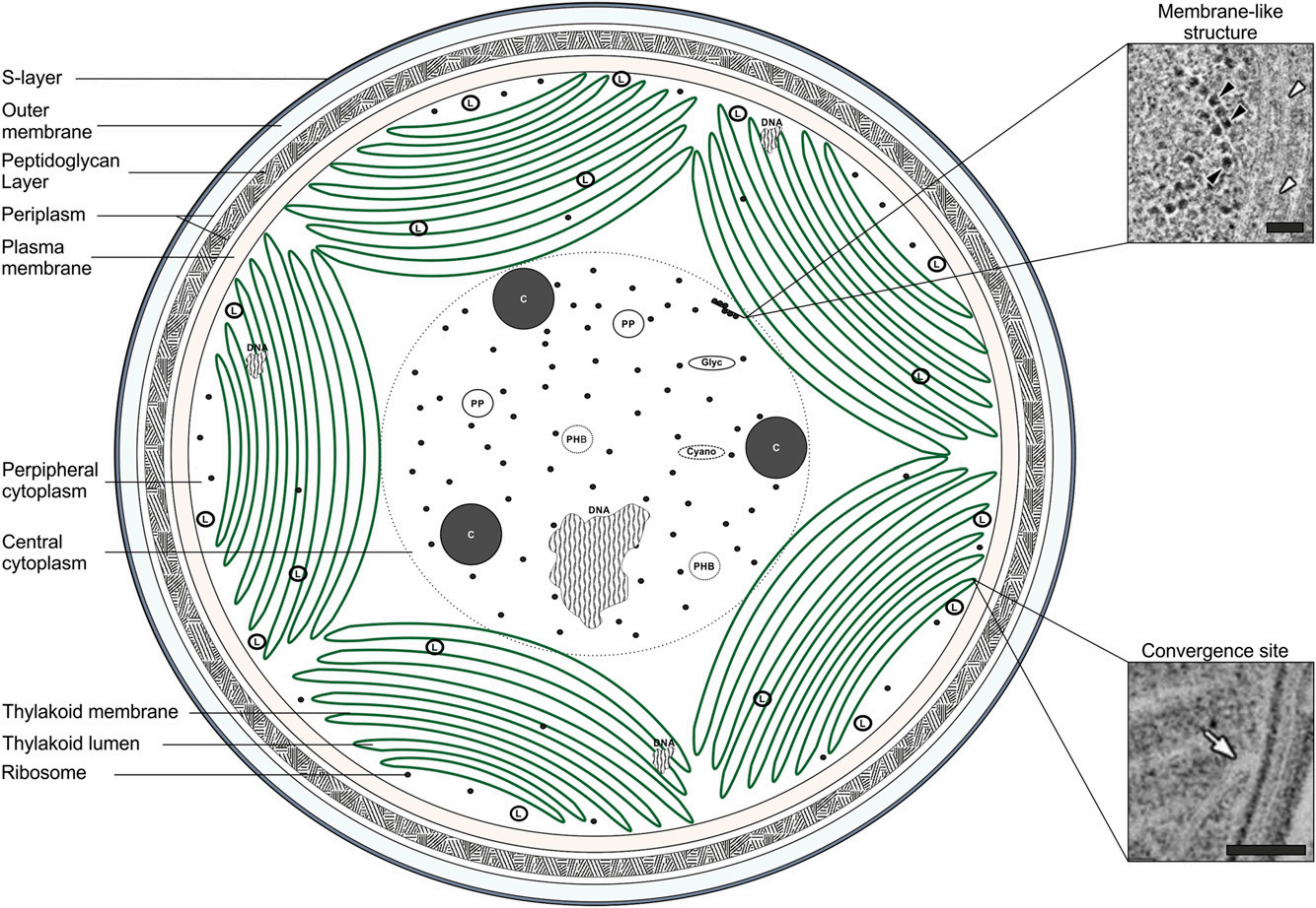
The ultrastructure of *Synechocystis* showing various subcellular components. L, Lipid body; C, Carboxysome; PHB, Polyhydroxybutyrate granule; PP, Polyphosphate body; Glyc, Glycogen granule; Cyano, Cyanophycin granule. SEMs taken from Van de Meene et al. (2016). Membrane-like structure in close association with ribosomes (black arrowhead) and seemingly continuous with TM (white arrowhead) are shown. Convergence site of the PM and TM (white arrow). Bars = 50 nm.

Cytoplasmic compartments such as the carboxysome, a proteinaceous structure in which carbon fixation occurs, and various storage bodies containing glycogen, cyanophycin, polyhydroxybutyrate (PHB), lipids, and polyphosphate, add further complexity to the cell (Liberton et al., 2006; van de Meene et al., 2006). Many species also contain multiple chromosomal copies (Griese et al., 2011), and in the case of the model cyanobacterium, *Synechocystis* sp. PCC 6803 (*Synechocystis*), ∼70% of ribosomes are localized in the central cytoplasm with the remainder in the cytoplasmic periphery between the PM and TM (20%) or within the TM stacks (10%; van de Meene et al., 2006).

Given this intricate organization, characterizing the distribution of the subcellular proteome is critical in understanding the biochemical and physiological processes within the cell and the role of individual cellular components, as their spatial organization will reflect protein function (Dreger, 2003). Moreover, the chloroplasts of algal and plant cells are descended from an internalized cyanobacterium (Howe et al., 2008), with many cyanobacterial genes (De Las Rivas et al., 2002; Martin et al., 2002) and structural features (Hinterstoisser et al., 1993) conserved in photosynthetic eukaryotes (Fig. 2). Therefore, knowledge of cyanobacterial protein localization will help in understanding the evolution of chloroplast ultrastructure from its cyanobacterial ancestors.

**Figure 2.**
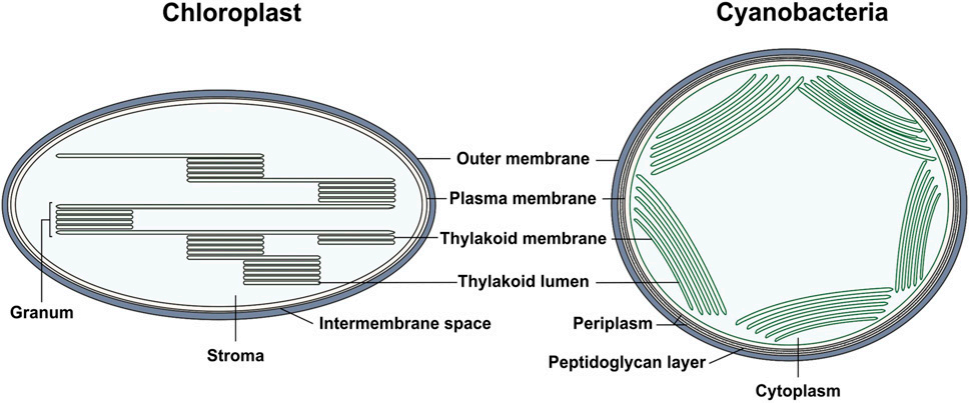
Structural similarities between cyanobacteria and chloroplasts. Schematic depictions of the similar membrane organization within a cyanobacterial cell and chloroplast are shown.

Multiple studies have attempted to verify the distribution of proteins in cyanobacteria, via analysis of isolated cellular fractions. This approach has been used to elucidate the proteomes of the membranous (Wang et al., 2000; Huang et al., 2002; Herranen et al., 2004; Huang et al., 2004; Srivastava et al., 2005; Huang et al., 2006; Pisareva et al., 2007; Wang et al., 2009; Zhang et al., 2009; Agarwal et al., 2010; Rowland et al., 2010; Wegener et al., 2010; Pisareva et al., 2011; Li et al., 2012; Plohnke et al., 2015; Liberton et al., 2016) and soluble (Simon et al., 2002; Huang et al., 2006; Kurian et al., 2006a, 2006b; Slabas et al., 2006; Suzuki et al., 2006; Zhang et al., 2009; Rowland et al., 2010; Wegener et al., 2010; Plohnke et al., 2015) compartments that constitute *Synechocystis* (Supplemental Table S1). In these studies membranes were typically isolated using two-phase aqueous polymer partitioning and/or Suc density ultracentrifugation, followed by gel based or shotgun proteomic analysis.

This approach has been applied to investigate PM (Huang et al., 2002; Pisareva et al., 2007; Pisareva et al., 2011; Liberton et al., 2016), TM (Wang et al., 2000; Srivastava et al., 2005; Agarwal et al., 2010; Pisareva et al., 2011; Liberton et al., 2016), OM (Huang et al., 2004), and soluble fractions (Simon et al., 2002). However, there are numerous inconsistencies in the assignment of protein localization to subcellular fractions between these studies (Srivastava et al., 2005; Pisareva et al., 2007; Pisareva et al., 2011; Liberton et al., 2016), suggesting that this approach of membrane fractionation could have limitations due to technical difficulties in separating cellular compartments and/or the complicated organization of cyanobacterial cells (Pisareva et al., 2011). For example, these methods have been shown to give ‘purified’ PM fractions that actually contain detectable amounts of TM (e.g. Zhang et al., 2015; Lea-Smith et al., 2016b). In addition, isolating membranes via two-phase aqueous polymer partitioning results in considerable losses of cellular material and under-sampling of the proteome. Furthermore, both the PM and TM may be heterogeneous (Srivastava et al., 2006; Agarwal et al., 2010; Pisareva et al., 2011) and previous work has suggested that only a hydrocarbon-rich fraction of the TM, and not the whole membrane, is purified via two-phase partitioning (Lea-Smith et al., 2016b). For example, a highly curved ‘convergence membrane’ substructure in the TM was recently observed, which was in close contact with the PM, and may play a role in biogenesis of thylakoid proteins (Rast et al., 2019).

Recently, a study was published by Liberton et al. on the distribution of proteins between the PM and TM in *Synechocystis* (Liberton et al., 2016). Two-phase separation was used to separate the cellular membranes into two partitions representative of the PM and TM. Proteins within these two fractions were then labeled using isobaric tags and analyzed via mass spectrometry (MS), resulting in the quantification of 1,496 proteins. Looking at the distribution of proteins across the two phases, the authors were able to assign 459 and 176 proteins to the PM or TM, respectively. This study eliminated the need to obtain complete purification of either membrane. However, much of the cellular material was discarded during the purification stages, and the simplified approach of partitioning into two phases meant that other subcellular compartments, such as the OM, the soluble proteins from the cytosol, thylakoid lumen, and periplasmic space, the carboxysome and storage bodies, were not taken into account. Additionally, the method was insensitive to proteins residing in multiple compartments. Furthermore, quantitative variation within the biological replicates, noted by the authors, rendered the dataset limited in its utility to assign membrane proteins to specific subcellular structures.

In this study we adapted the hyperLOPIT approach to map the proteins of the entire *Synechocystis* cell using spatial proteomics applied to cellular fractions enriched with various subcellular membranes (Mulvey et al., 2017; Thul et al., 2017). This method relies on the correlation of proteins within these subcellular fractions using stable isotope tagging coupled with machine learning approaches to assign similar fractionation behavior. The output of this method is the steady state location of a protein within a cell. This approach resulted in the identification of 2,445 proteins. This study provides the most complete description of the *Synechocystis* proteome to date, covering ∼67% of the predicted proteome, and assigns 1,712 proteins to specific regions of the cell, which can be interrogated via an interactive database. These regions include the PM, TM, small and large ribosomal subunits, PHB storage body and soluble fraction, adding a further layer of complexity when compared with previous studies. This work uses a simplified strategy to separate the contents of the cell, overcoming problems in the purification of membrane systems and loss of cellular components, leading to a more thorough understanding of the spatial distribution of proteins within a cyanobacterial cell.

For interactive data mining and data visualization, we have deployed a dedicated online data app for the community at https://lgatto.shinyapps.io/synechocystis/. The app contains a searchable and clickable data table, visualization of the quantitative protein profiles across both replicates, and a fully interactive principal component analysis (PCA) plot.

## RESULTS

### Fractionation of *Synechocystis* Cell Extracts by Suc Density Ultracentrifugation

In order to fractionate cellular components, *Synechocystis* cells were cultured to late-logarithmic phase (Supplemental Fig. S1) under continuous moderate light (60 µmol photons m^−2^ s^−1^) with air-bubbling at 30°C. Growth conditions and cell harvesting are similar as those performed in studies where membranes were isolated using two-phase aqueous polymer partitioning (e.g. Norling et al., 1998; Pisareva et al., 2007), allowing a comparison of protein localization between these datasets. Cells were subsequently lysed and the extract fractionated via Suc density centrifugation (Schottkowski et al., 2009). Separation on a step gradient resulted in cellular material accumulating in the heaviest fraction (Supplemental Fig. S2A).

**Figure 3.**
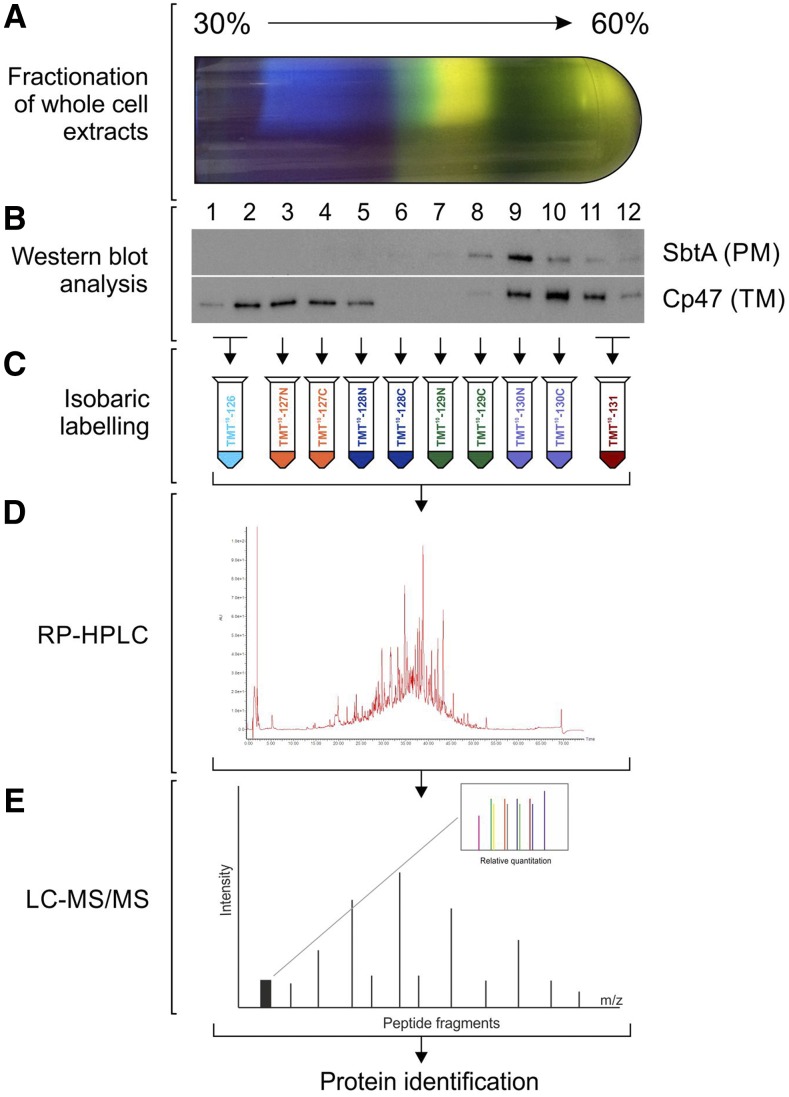
Outline of the proteomic workflow. A, Total protein was extracted from each of the gradient fractions and quantified. B, The different distributions of TM and PM, as indicated by immunoblot analysis using antibodies against TM (CP47) and PM (SbtA) specific marker proteins C, Fractions 1–2 and 11–12 were merged to yield 10 gradient fractions and each labeled with a different tag using a 10-plex TMT kit. These fractions were merged as they exhibited similar protein profiles according to SDS-PAGE and immunoblot analysis. D, Reverse phase-HPLC was used to separate the proteins according to their hydrophobicity. E, This provided better resolution before subsequent MS/MS analysis. Proteins were identified by comparison with the database held by CyanoBase, and quantified using Proteome Discoverer Software 1.4.1.14 (Thermo Fisher Scientific).

Further separation of this fraction on a continuous Suc gradient was therefore required. This resulted in 12 fractions with varying protein-pigment composition (Fig. 3A), as determined by absorption spectra measurements (Supplemental Fig. S2B), diverse protein profiles, as evaluated by SDS-PAGE (Supplemental Fig. S2C), and different distributions of TM and PM, as indicated by immunoblot analysis using antibodies against TM (PSII core light harvesting protein [PsbB {CP47}]) and PM (sodium-dependent bicarbonate transporter [SbtA])-specific marker proteins (Fig. 3B). These results demonstrate the validity of this approach in effectively separating and enriching cellular components, a necessary prerequisite for labeling and subsequent analysis.

### Extensive Coverage of the *Synechocystis* Proteome by Mass Spectrometry Reveals Subclustering of Different Compartments

Of the twelve fractions obtained from the continuous Suc gradient, both the lightest two and the heaviest two were deemed to be most similar to one another compared with other fractions by SDS-PAGE and were thus combined in pairs to yield ten fractions, reflecting the number of Tandem Mass Tags (TMT) tags in a 10-plex reagent set. These ten fractions were then labeled with the TMT reagents (Fig. 3C). Reverse phase-HPLC was used to separate the proteins according to their hydrophobicity (Fig. 3D) and provide better resolution before subsequent MS/MS analysis (Fig. 3E). In total, the MS analysis resulted in the identification of 2,445 proteins (Supplemental Tables S2and S3) across both biological replicates, out of a potential 3,672 listed in the CyanoBase database (http://genome.annotation.jp/cyanobase). This included 397 predicted integral membrane, 768 hypothetical and 400 unknown proteins.

Similar scale proteome coverage (2,461 proteins) was recently reported by Spät et al. (2018). In their study MS analysis was performed on cells cultured under similar environmental conditions (40 µmol photons m^−2^ s^−1^ with air-bubbling at 26°C) to those used here, but which were nitrogen deprived and then harvested 2, 8, 24, and 55 h after resuscitation via addition of nitrate. A comparison of protein coverage between our data and Spät et al. showed that 2,127 proteins (∼58%) were detected in both studies (Supplemental Table S4), suggesting that this may be the core proteome expressed under these laboratory conditions. Only 318 proteins were detected in our study (Supplemental Table S5), whereas 334 were unique to Spät et al. (Supplemental Table S6). These differences are likely due to the physiological response induced during resuscitation from nitrogen deprived to replete media or variation in cell preparation and proteome detection methods. Moreover, only 109 proteins were detected in some of the five Spät et al. samples, and 82 were detected at very low quantities. Also, 856 (∼25%) were not detected in either study (Supplemental Table S7), which included 112 with transposon related functions, 290 hypothetical, and 275 unknown proteins. This portion of the proteome may be dormant under these laboratory conditions.

**Figure 4.**
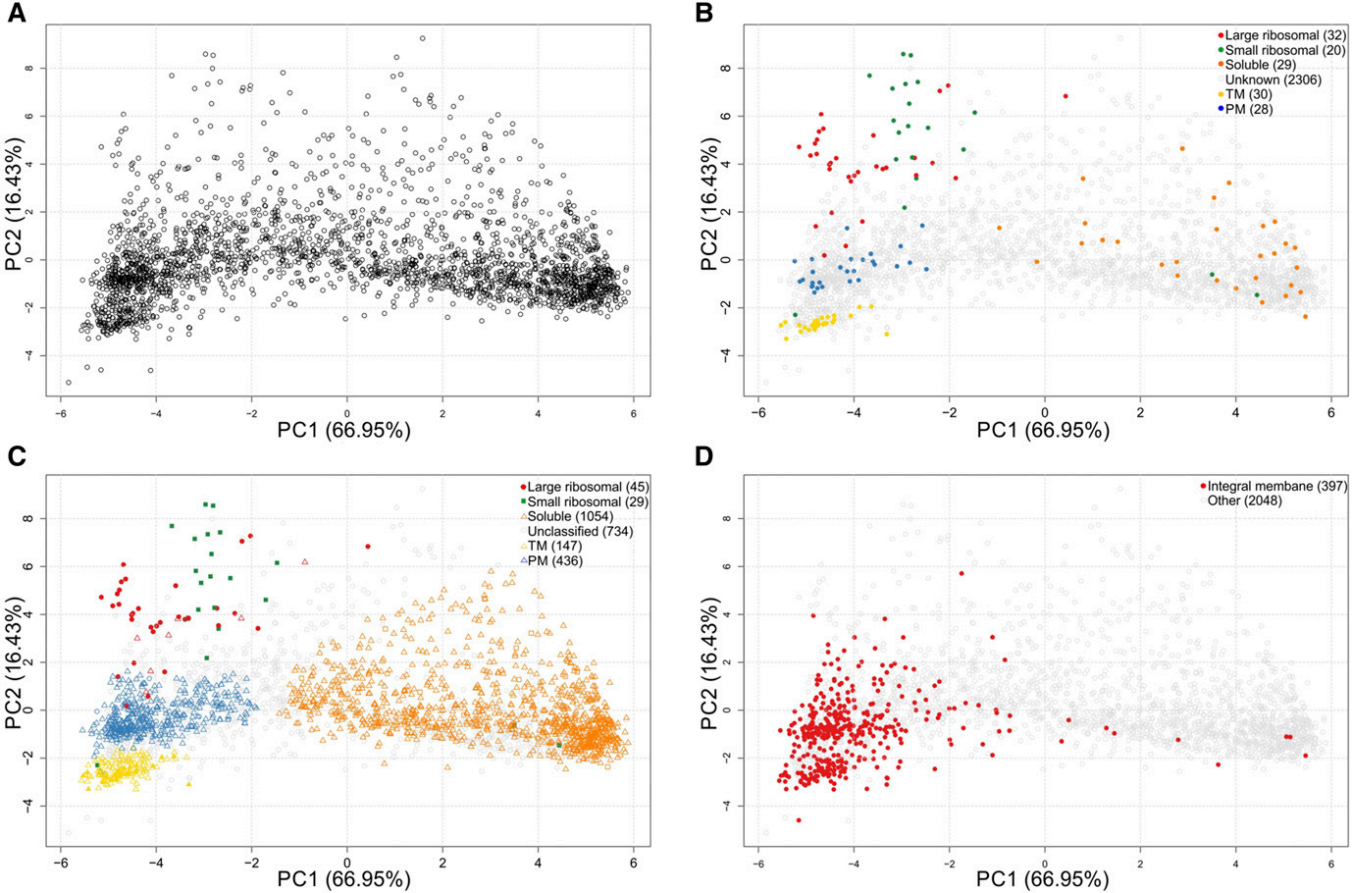
PCA plots. A, PCA of the combined biological replicates. B, PCA plot showing the location of protein markers. C, PCA plot showing the assignment of proteins to subcellular regions. A cutoff of 0.75 (corresponding to 75%) was used for the boundaries of the TM, PM, small and large ribosomal subunits, and 0.65 for the soluble proteins. Gray circles indicate proteins with an unclassified localization. D, Integral membrane proteins highlighted on the PCA plot of combined datasets.

In order to localize proteins to specific regions of the cell, the abundance profile of each protein along the Suc gradient was first quantified using the distribution of TMT reporter ions generated by tandem MS. Assuming that proteins which reside together in the cell would cofractionate in the Suc gradient, we therefore used this data to interpret the distribution of proteins within the cell. Resulting abundance profiles of proteins were subjected to PCA for visualization purposes. The PCA plot represents a map of all 2,445 proteins identified in both biological replicates, in which proteins with similar distribution profiles along the gradient are clustered together (Fig. 4A). Marker proteins for subcellular compartments, including the PM and TM, small and large ribosomal subunits, and soluble proteins (including cytosolic, thylakoid lumen, and periplasmic proteins; Fig. 4B; Supplemental Table S8) were used to identify which clusters on the plot correspond to which subcellular regions. This resulted in identification of distinct clusters corresponding to certain subcellular regions, including the PM, TM, small ribosomal subunit, large ribosomal subunit, and soluble proteins, without the need to obtain pure membrane fractions.

The localization of previously unclassified proteins was achieved by matching their profiles along the Suc gradient to the marker protein profiles. This was carried out using supervised classification with a support vector machine (SVM; Gatto et al., 2014) to assign unclassified proteins, defining the boundaries of the subcellular regions (Fig. 4C), and producing an SVM score for each protein and a predicted localization. The SVM score is a measure of the confidence with which the protein was classified. The majority of assigned proteins (1,054) were found to be soluble, followed by those that were localized to the PM (436) or TM (147), with only a small number associated with the small (29) and large (45) ribosomal subunits, including the protein markers themselves (Supplemental Table S3). No integral membrane proteins localized to the soluble fraction (Fig. 4D), although a large number of proteins lacking transmembrane helical domains (Supplemental Table S3) localized to the PM and TM. The remaining 734 proteins were not classified into any of these subcellular locations, and were thus given an ‘unclassified’ allocation. Of the 1,168 unknown and hypothetical proteins, 56 were TM localized, 233 PM localized, and 467 were found to be soluble. Seven and five proteins were associated with the small and large ribosomal subunit fractions, respectively. Further description of the localization of sets of proteins including those with a previously assigned function is given in detail in the supplemental information, along with comparisons with published localization information.

**Figure 5.**
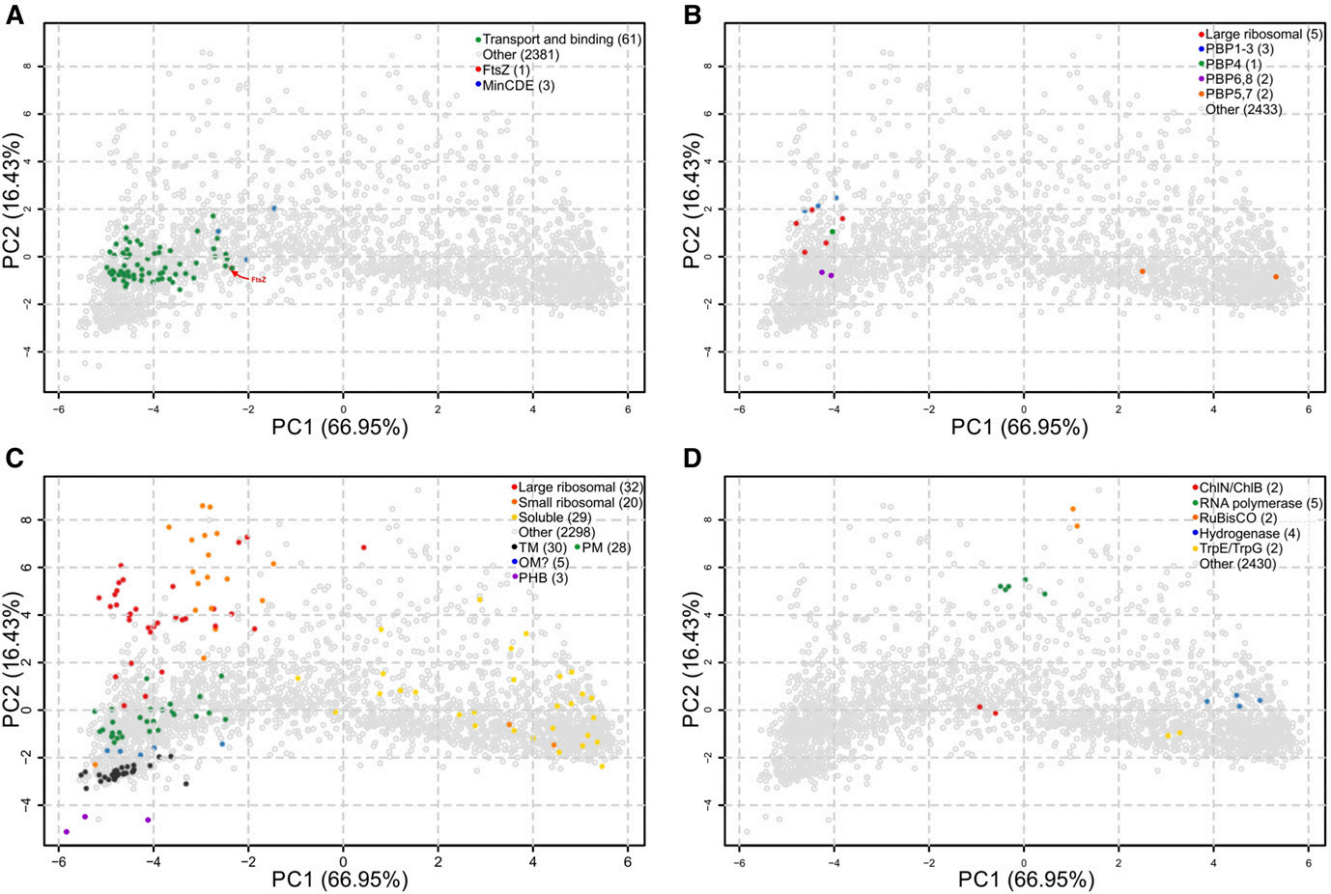
Clustering of proteins with similar functions indicates potential further subcellular regions and compartmentalization. A, Two distinct subclusters of transport and binding proteins can be seen within the PM region. The smaller of these two groups is in close proximity to FtsZ, which forms the septal ring, and the MinCDE proteins, which control the position and shape of the spectral ring. B, Subclustering of certain large ribosomal subunit proteins was observed, in close association with PBP1-3 to the PM region. The location of PBP4-8 are shown. C, Proteins thought to reside in the OM were found to localize to a distinct and unclassified region in between the PM and TM regions. Proteins involved in PHB biosynthesis are highlighted in purple. D, Numerous proteins that form complexes were found in very close proximity to each other on the PCA plot.

Further subcellular regions and compartmentalization within the cell were observed. For example, the PM proteome grouped into two distinct regions (Figs. 4C and 5A). A small proportion of transport and binding proteins were sublocalized within the PM cluster, in close association with the cell division protein FtsZ, which forms the septal ring, and the MinCDE proteins, which control the position and shape of the septal ring. Large ribosomal subunits also grouped into two distinct regions with five proteins (L16, L28, L27, L19, and L35) forming a distinct cluster close to the PM region (Fig. 5B). This region also contains the high *M*_r_ Class A penicillin binding proteins (PBPs) PBP1-3, thought to operate in cell elongation and cytokinesis (Marbouty et al., 2009b). Although little is known about the OM proteome, four proteins designated as ‘probable porin; major OM proteins’ by CyanoBase, and PilQ, the OM subunit of the pili, were grouped together in a distinct cluster between the PM and TM regions (Fig. 5C). Moreover, the subunits of certain complexes clustered together. These included RNA polymerase, Rubisco, and hydrogenase, as well as complexes involved in chlorophyll (light-independent protochlorophyllide reductase subunits ChlN/ChlB) and Trp/folate biosynthesis [anthranilate synthase component I/II (TrpE/TrpG); Fig. 5D]. This indicates that some complexes are not disassociated by cell rupture and Suc gradient separation of cellular contents.

### Comparison with Previous Subcellular Localization Data for the *Synechocystis* Proteome

Of the previous studies on subcellular distributions of *Synechocystis* proteins, the most comprehensive list was achieved by Liberton and coworkers who used quantitative proteomics coupled with two-phase separation of cellular membranes to determine the protein content of the PM and TM (Liberton et al., 2016). Supplemental Figure S3A shows the comparison of the Liberton data with those presented here. Of note, where both studies assign a protein to either the PM or TM, there is a high degree of overlap between the assignment and very few proteins assigned to the PM by Liberton et al. are assigned to the TM in this study and vice versa. There is only limited overall overlap between TM assignments and PM assignments, however, between the two studies (Supplemental Fig. S3B). This is in part due to the facts that different proteins were identified in both studies and that the study presented here represents the whole cell, whereas the Liberton study analyzed only a subset of proteins. Many proteins thought to be TM or PM localized by the Liberton study are not assigned to either membrane here. It is not clear whether the additional PM and TM proteins presented in the Liberton study represent contamination of their TM and PM enriched fractions with proteins from other parts of the cell, or that the lack of overlap is a result of the fact that the study presented here returns the steady state location of proteins. Hence, if a TM and PM protein were also elsewhere in the cell, our study would flag it up as ‘mixed location.’ It is interesting to note that many of the results for the TM and particularly the PM in Liberton’s study are assigned to the soluble protein set in the data presented here, demonstrating the importance of mapping the whole cell and not just isolated fractions. Analysis of these proteins shows that only 7% have a predicted single transmembrane domain, and the remainder have no predicted membrane spanning regions; therefore, a location in the TM or PM seems less likely.

### Metabolic Pathways Are Distributed Throughout the Cell

**Figure 6.**
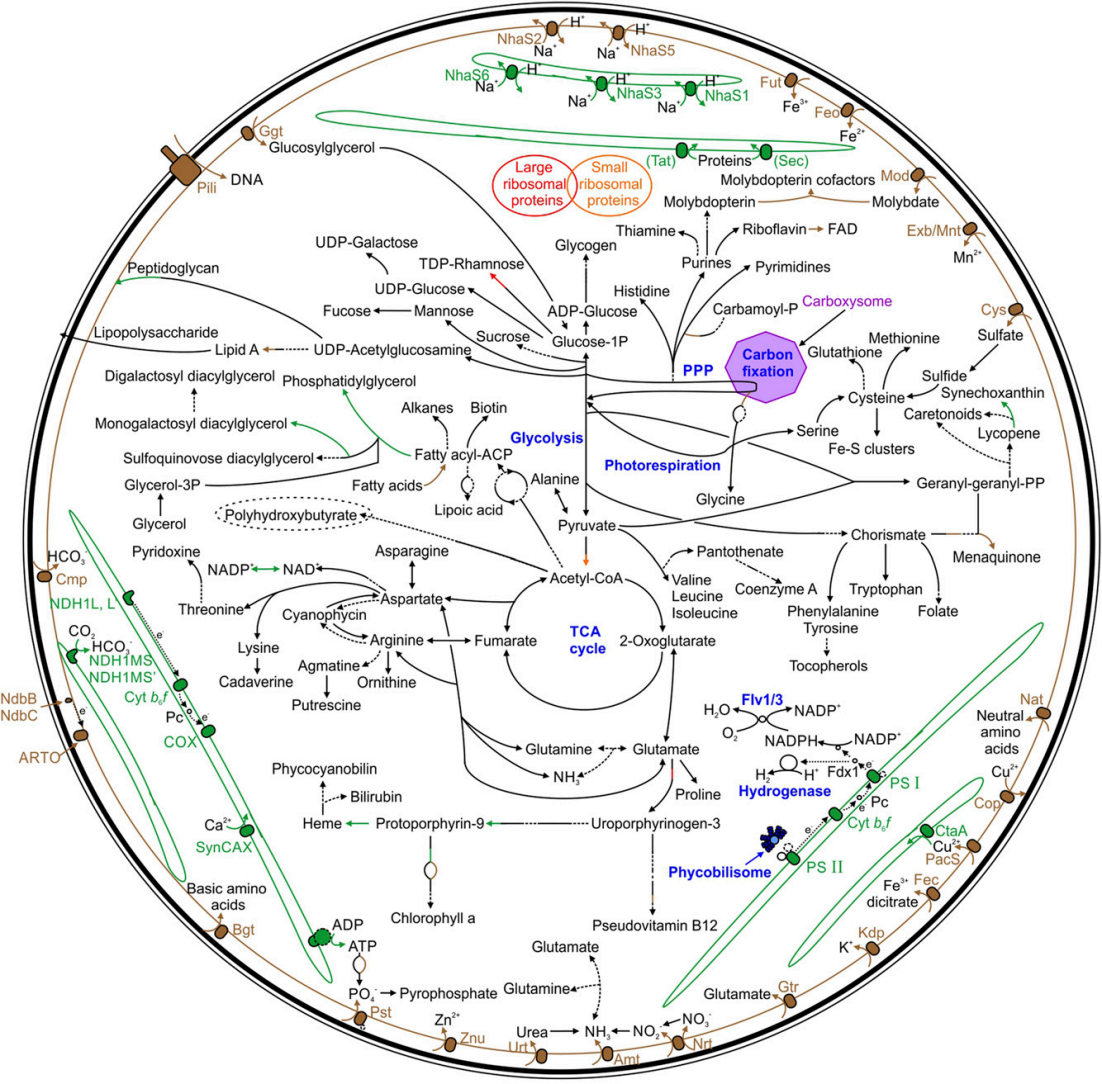
Predicted localization of proteins and biosynthetic pathways in *Synechocystis*. Enzymatic steps within a pathway, which are localized to different regions of the cell, are separated into appropriate colors/styles. Green, TM; Brown, PM; Solid line, Soluble; Broken line, unclassified; TCA cycle, tricarboxylic cycle; PPP, pentose phosphate pathway; Flv 1/3, Flavodiiron protein 1/3. Refer to Supplemental Table S3 for protein abbreviations.

Enzymes involved in metabolism predominantly localized to the soluble region, including those synthesizing amino acids, cofactors, prosthetic groups and carriers, glycolysis, tricarboxylic acid cycle and pentose phosphate pathway intermediates, cell wall components, purines and pyrimidines, fatty acids, phospholipids, sterols, and hydrocarbons (Fig. 6; Supplemental Table S3). However, some enzymes, predominantly those involved in the final catalytic steps of certain metabolites, localized to membranes. These included enzymes synthesizing membrane lipids (acyltransferase PlsC, fatty acid/phospholipid synthesis protein PlsX, monogalactosyl diacylglycerol synthase MgdA and phosphatidate cytidylyltransferase CdsA), all of which localized to the TM. This is likely due to the thylakoids constituting the bulk of the membranes in the cell, and it is possible that a minor percentage of these proteins are PM localized.

Other TM localized enzymes include those synthesizing heme (ferrochelatase HemH, protoporphyrinogen IX oxidase HemJ) and transhydrogenation of NADP^+^ (PntA, PntB). HemJ converts protoporphyrinogen IX to protoporphyrin IX, the precursor of heme and chlorophyll (Skotnicová et al., 2018). A recent study in *Chlamydomonas reinhardtii* indicates that HemJ likely requires plastoquinone as an electron acceptor (Brzezowski et al., 2019). Localization of HemJ to the TM in *Synechocystis* suggests a similar enzymatic reaction is possible. TM localization of PntA/B is consistent with the majority of NADP^+^ undergoing reduction to NADPH via ferredoxin-NADP reductase in the TM photosynthetic electron transport chain, and heme acting as a precursor for phycobilins, subsequently incorporated into phycobilisomes.

Enzymes synthesizing phylloquinol (2-phytyl-1,4-benzoquinone methyltransferase MenG, MenH), FAD (RibF), and molybdopterin cofactors (MoeA) were associated with the PM. It is unclear why RibF is PM localized. MenG is closely associated with the type two NAD(P)H dehydrogenase, NdbB, on the PCA plot. Both proteins are required for the final biosynthetic step of phylloquinol biosynthesis, and their close association suggests they may form a complex (Fatihi et al., 2015). PM localization of MoeA may aid incorporation of imported molybdate into the molybdopterin cofactor.

In addition, several enzymes catalyzing carotenoid biosynthesis localized to the membranes. Carotenoids play a key role in assembly of photosynthetic complexes (Tóth et al., 2015), membrane integrity, and thylakoid organization (Mohamed et al., 2004), and as light harvesting and photoprotective pigments. Seven carotenoids have been detected in *Synechocystis*: synechoxanthin, myxol-2’-dimethylfucoside (myxoxanthophyll), zeaxanthin, 3′-hydroxy-echinenone, cis-zeaxanthin, echinenone and β-carotene (Graham and Bryant, 2008). Carotenoids have been localized to both membrane fractions (Zhang et al., 2015), but the enzymes involved in biosynthesis of these compounds have not been completely elucidated or their intracellular location determined (the pathway is detailed in Supplemental Fig. S4). Enzymes involved in γ-carotene (CruF) and β-carotene (CrtL and CruA) biosynthesis (Maresca et al., 2007) were TM localized, as were the only enzymes identified in synechoxanthin (CruE, CruH) and myxoxanthophyll (CruG) biosynthesis (Graham and Bryant, 2009). The only carotenoid biosynthetic enzyme localized to the PM was the carotene isomerase CrtH, involved in cis-to-trans conversion of carotenes (Masamoto et al., 2001). However, carotenoid biosynthesis in a ΔCrtH mutant is only affected under dark conditions, not light, and its exact role in the cell has not been determined (Masamoto et al., 2001).

A few proteins involved in intermediate enzymatic steps localized to membranes. For example, the long-chain-fatty-acid CoA ligase Aas, involved in the cycling of free fatty acids via activation by acyl carrier protein (ACP), localized to the PM, which is in agreement with Liberton et al. (2016). This supports the proposed role of Aas in mediating fatty acid import (von Berlepsch et al., 2012). Dihydroorotate dehydrogenase (PyrD), the only membrane associated enzyme involved in nucleotide metabolism, also localized to the PM. In *E. coli*, PyrD requires a respiratory quinone as an electron acceptor (Nørager et al., 2002). Our data suggest that *Synechocystis* PyrD may use plastoquinone as an electron acceptor, which could be one of the roles of the PM electron transport chain.

### Assembly of Cell Wall Components Occurs in Both Membranes

**Figure 7.**
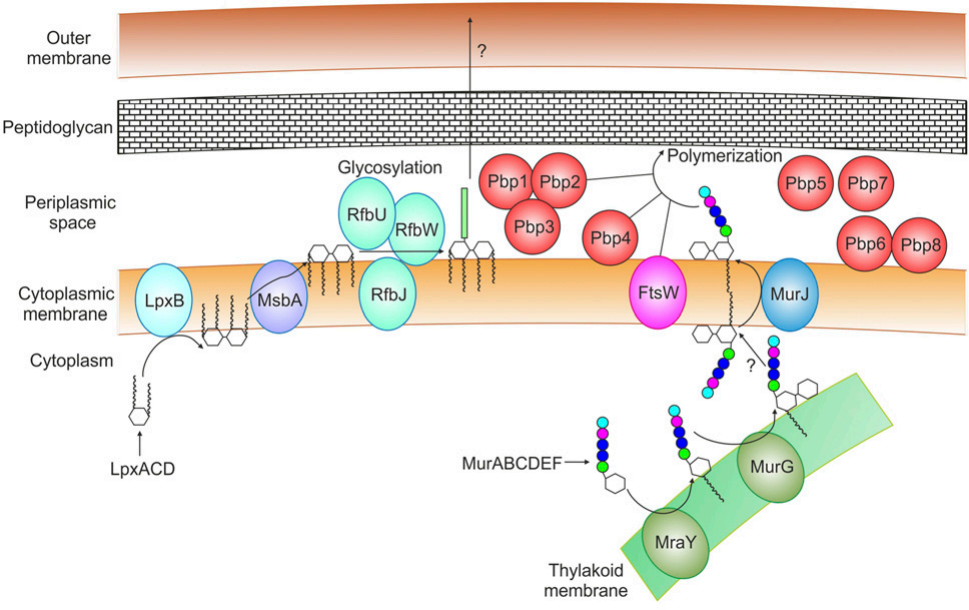
Schematic diagram detailing biosynthesis of lipopolysaccharides (LPSs) and assembly and polymerization of peptidoglycan (PG) monomers. LpxACDB enzymes synthesize the LPS disaccharide precursor. In *E. coli*, the flippase MsbA transfers the disaccharide to the periplasmic side of the PM, although the cyanobacterial MsbA has not been identified. RfbJUW are hypothesized to glycosylate the disaccharide. The LPS is transported to the OM by an uncharacterized protein complex. PG monomers are synthesized by MurABCDEFG and MraY enzymes. Localization of MraY and murG in the TM suggests that the monomers are subsequently transported to the PM, where the flippase, MurJ, transfers the monomers to the periplasmic side. Penicillin binding proteins Pbp1-4 and FtsW are involved in PG polymerization, whereas Pbp5-8 are likely involved in PG depolymerization. A question mark indicates uncharacterized processes.

A similar pattern was observed with enzymes involved in biosynthesis of cell wall components (Fig. 7). The enzymes catalyzing the initial steps of the core region of lipopolysaccharides (LpxACD) were soluble, whereas the one catalyzing the final step of lipid A disaccharide bisynthesis (LpxB), localized to the PM. MsbA, the flippase that translocates lipid A disaccharide across the PM (Ruiz et al., 2009), has not been identified in cyanobacteria. However, four genes with high sequence similarity to *E. coli msbA* (*slr2019, sll1276, sll1725, slr1149*; 70.5%, 69.6%, 64.9%, 66.3% similarity, respectively) were identified in our study. All localized to the PM; therefore, further genetic and biochemical studies will be required to identify cyanobacterial MsbA. Several putative glycosyltransferases (RfbU, 2 × RfbW, RfbJ, RffM), postulated to add sugar groups to the outer core of this molecule (Fisher et al., 2013), also localized to the PM. Homologs of the proteins in the Lpt transport complex, responsible for transporting lipopolysaccharides from the PM to the outer leaflet of the OM in *E. coli* (Ruiz et al., 2009), are not present in *Synechocystis*, suggesting an alternate system must perform this role.

The enzymes catalyzing the initial steps of peptidoglycan monomer biosynthesis (MurABCDEF) were soluble. Somewhat suprisingly, the final two steps of peptidoglycan monomer biosynthesis (MraY, MurG) localized to the TM, not the PM as would be expected. MurG has been identified as TM specific in a previous study (Pisareva et al., 2011). This would suggest that monomers are assembled at the TM, and subsequently transported to the PM. A single homolog of MurJ (slr0488), the flippase that translocates peptidoglycan monomers across the PM (Sham et al., 2014), is present in *Synechocystis* but was not detected in our study or in Spät et al. or Liberton et al. (Liberton et al., 2016; Spät et al., 2018). Neither was FtsW, responsible for peptidoglycan polymerization in association with PBPs (Taguchi et al., 2019). Our knowledge of the role of cyanobacterial PBPs is limited, although all eight putative PBPs, separated into class A (PBP 1-3), B (PBP4/FtsI), and C (PBP 5-8), were detected. Although PBP4 is essential in *Synechocystis*, single mutants deficient in one class A or C PBP have been generated, although not mutants lacking two of each class (Marbouty et al., 2009b). PBP1-3 colocalized in a unique cluster on the PCA plot, PBP4, and PBP6/8 localized to different PM regions, whereas PBP5/7 was soluble (Fig. 5B). Both class A and B PBPs are believed to be involved in peptidoglycan polymerization, with class A primarily involved in synthesis of the cell wall linked to cell elongation, whereas class B interacts with other proteins of the divisome, with a primary role in cell division (Sauvage et al., 2008). Other components of the divisome including Cdv3, ZipN, and ZipS (Marbouty et al., 2009a) also localized to the PM in our study. In *Synechocystis*, the Type C PBPs are divided into two classes, type 4 (PBP 5/8) and AmpH (PBP 6/7; Marbouty et al., 2009b). PBP5/7 are soluble, presumably in the periplasm, whereas PBP6/8 are PM associated. Their primary role is likely in disassembling the peptidoglycan heteropolymer with other proteins such as the *N*-acetylmuramoyl-l-Ala amidases, which were also PM localized (Slr1744) or soluble (Slr0891; van Heijenoort, 2011).

### The Thylakoid Membrane Proteome Is Predominantly Involved in Energy Conversion

**Figure 8.**
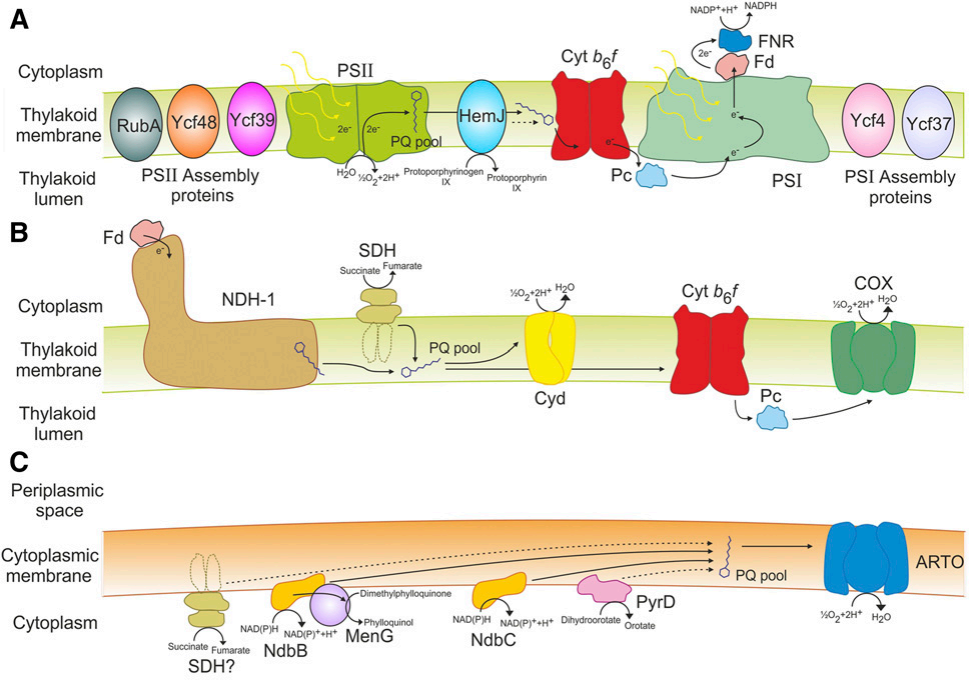
Schematic diagram detailing localization of the electron transport complexes in cyanobacteria. Shown are the thylakoid membrane photosynthetic (A) and respiratory (B) electron transport chains, and the plasma membrane electron transport chain (C). PQ, plastoquinone; HemJ, protoporphyrinogen IX oxidase; cyt *b*_6_*f*, cytochrome *b*_6_*f*; Pc, plastocyanin; Fd, ferredoxin; FNR, ferredoxin-NADP+-reductase; NDH-1, NDH dehydrogenase 1; SDH, succinate dehydrogenase; Cyd, bd-quinol oxidase; COX, cytochrome-c oxidase; NdhB, NAD(P)H dehydrogenase 2 B; NdbC, NAD(P)H dehydrogenase 2 C; MenG, Demethylphyloquinone methyltransferase; PyrD, dihydroorotate dehydrogenase; ARTO, alternative respiratory terminal oxidase. Also shown are the PSII assembly proteins RubA (Rubredoxin A), Ycf48 and Ycf39 and the putative PSI assembly proteins Ycf4 and Ycf37. Localization of SDH in the PM has not been confirmed. Dotted lines indicate possible electron transport routes.

As expected, the majority of subunits in photosynthetic complexes, including PSI and PSII, and cytochrome *b*_6_*f* (cyt *b*_6_*f*), were TM localized (Fig. 8A; Supplemental Table S3). Other proteins associated with photosystems including the PSII assembly protein RubA, Ycf48, and Ycf39 (García-Cerdán et al., 2019; Kiss et al., 2019), the putative PSI assembly proteins Ycf4 and Ycf37, and IsiA, which is required for PSI formation and state transitions under iron starvation, were also TM localized. In addition, CpcG2, an integral protein of the phycobilisome, the light harvesting complex of cyanobacteria, localized to this compartment, although other phycobilisome subunits were predominantly soluble. Respiration has previously been established to occur in the TM (Lea-Smith et al., 2016a), although the location of electron transport complexes has not been fully established. Of the respiratory electron donors, only NADH dehydrogenase type 1 subunits were TM localized (Fig. 8B). The membrane subunits of succinate dehydrogenase have not been identified (Lea-Smith et al., 2016a), although it has been suggested as the main TM localized respiratory donor (Cooley and Vermaas, 2001). Subunits of two terminal oxidases, cytochrome-*c* oxidase and cytochrome *bd*-quinol oxidase, localized to the TM. Interestingly, ATP synthase subunits localized to the TM, in agreement with Liberton et al. (Liberton et al., 2016). Overall, this suggests that energy conversion is predominantly localized to the TM. Other proteins of note that localized to the TM include three FtsH proteins involved in PSII repair (FtsH2, FtsH3, FtsH4), the thiol:disulphide interchange protein TrxA, and the detoxification protein Slr0236. Only six proteins involved in transport localized to the TM, including three Na^+^/H^+^ antiporters (NhaS1, NhaS3, NhaS6), the copper importer CtaA, the H^+^/Ca^2+^ exchanger SynCAX, and an ABC transporter (Sll0759). Of the 83 characterized proteins localized to the TM, 63 are involved in energy conversion, photosystem repair/assembly, or synthesis of lipids required for membrane assembly or photosystem function.

### The Plasma Membrane Proteome Is Predominantly Involved in Transport and Regulatory Functions

The majority of proteins involved in transport localized to the PM (Fig. 6; Supplemental Table S3). These included the transporters of ammonium, basic and neutral amino acids, Glu, bicarbonate, inorganic iron and iron dicitrate, glucosylglycerol, manganese, molybdate, nitrate/nitrite, phosphate, potassium, sulfate, urea, and zinc. Copper is required in both the cytoplasm and thylakoid lumen. Previously it has been thought that copper is transported into the cytosol and thylakoid lumen via PM-localized CtaA and TM-localized PacS, respectively, based on studies performed in *Synechococcus elongatus* (Kanamaru et al., 1994; Tottey et al., 2012). In contrast, our results placed CtaA in the TM and PacS in the PM.

A second, poorly characterized, electron transport chain localizes to the PM (Lea-Smith et al., 2016a). Two NAD(P)H dehydrogenase type 2 electron donor proteins (NdbB, NdbC) and subunits of the alternative respiratory terminal oxidase localized to the PM, suggesting the presence of a simpler electron transport chain in this compartment (Fig. 8C). NdbB is required for phylloquinol biosynthesis (Fatihi et al., 2015). Deletion of NdbB resulted in almost a complete loss of phylloquinol and accumulation of the precursor molecule, 2-phytyl-1,4-naphthoquinone. NdbB was shown to reduce 2-phytyl-1,4-naphthoquinone to 2-phytyl-1,4-naphthoquinol using electrons derived from NADPH (Fatihi et al., 2015), which is subsequently methylated to phylloquinol by MenG (Sakuragi et al., 2002). Other proteins of note that localized to the PM included the cell division proteins MinD and FtsH1, the chaperone DnaK3, chemotaxis proteins PixJ1 and TaxD2, the competence protein ComE involved in DNA uptake, the detoxification protein Gst1 and the sigma factor SigF. Pili proteins localized to the PM, including 8/11 PilA designated subunits (another, PilA6, is unclassified but is in the PM region of the PCA plot), with the exception of PilQ, the OM subunit, and PilH, which was soluble. PilA1 is required for formation of thick pili (Yoshihara et al., 2001), but expression of the other 8 PilA proteins suggests they have a functional role in the cell under these growth conditions. Two proteins involved in DNA replication, DnaG, the DNA primase, which synthesizes oligonucleotides, and DnaX, a DNA polymerase II subunit, were both PM localized. The PM may therefore play an active role in DNA replication or regulation, which has been suggested to occur in *E. coli* (Saxena et al., 2013; Magnan et al., 2015).

### Protein Translocation Pathways Localize to the Thylakoid Membrane

The mechanism by which cyanobacteria target proteins to different membranes is poorly characterized. Single copy homologs encoding proteins involved in the Secretory (Sec), Twin-Arg Translocation (Tat), and Signal Recognition Particle (SRP) protein translocation pathways are present in the *Synechocystis* genome (Kaneko et al., 1996). Components of each pathway were either soluble or TM localized.

Two leader peptidases (LepB1, LepB2), which are involved in generation of mature proteins and may also have a role in releasing proteins into the correct compartment, have been identified in *Synechocystis*. Only LepB2 is essential for cell viability, and the two are not functionally redundant (Zhbanko et al., 2005). Both leader peptidases were identified in the study; LepB1 localized to the PM, whereas LepB2 was unclassified. In contrast with this work, previous proteomic studies and investigations into the leader peptidases have identified LepB1 as a TM-specific protein, with a suggested function in maturation of the photosynthetic machinery (Srivastava et al., 2005; Zhbanko et al., 2005; Pisareva et al., 2011; Liberton et al., 2016).

### Various Intracellular Organelles Localize to Distinct Regions of the Cytosol

Transmission electron microscopy indicates that carboxysomes in *Synechocystis* are located in the central cytoplasm (van de Meene et al., 2006). Most carboxysome subunits were found to be soluble, with the exception of CcmM, which was PM localized, and CcmN and CcaA, which were localized to an unclassified fraction. CcmM and CcmN are core shell proteins, and CcaA is the carbonic anhydrase, converting HCO_3_^−^ to CO_2_ (Gonzalez-Esquer et al., 2015). This suggests that certain subunits may interact with the PM or that cell disruption and subsequent separation caused the carboxysome to break apart due to its large size (between 80 and 150 nm in diameter), resulting in distribution of various subunits across the Suc gradient and in the PCA plot (Supplemental Fig. S5). Interestingly, the enzyme catalyzing the initial step of photorespiration (Pgp), the conversion of phosphoglycolate to glycolate, was also PM localized. The two subunits of Rubisco, RbcS and RbcL, which are assembled into the carboxysome (Wang et al., 2019), were found in a different area and grouped in a distinct unclassified fraction.

Of the enzymes involved in forming compounds that aggregate into storage bodies, only heterodimeric PHB synthase (PhaE/PhaC), catalyzing the final step of PHB biosynthesis, was found. PhaE/PhaC, along with PhaP (ssl2501), which is the surface coding protein of PHB granules, mapped to a unique unclassified region separate from any other proteins on the PCA plot (Fig. 5C). This suggests PHB synthesis may occur in a specific, distinct part of the cytosol (Hauf et al., 2015). GFP labeling of PhaC, PhaE, and PHB granules indicate that these biosynthetic steps are localized to the cell periphery (Hauf et al., 2013).

### Profiles of Ribosomal Subunits Show Clustering in a Specific Region of the PCA Plot

The majority of the large ribosomal subunit proteins localized to a specific fraction separate from the TM, PM, and soluble regions (Fig. 4C). Likewise, the majority of the small ribosomal proteins clustered in a specific region of the plot, distinct from the large ribosomal subunit protein area (Fig. 4C). However, three small ribosomal proteins were found in other locations on the plot. Two poorly characterized Rps1 homologs (Rps1A, Rps1B) localized to the soluble fraction, whereas Rps3 localized to the TM. Rps1 subunits are not present in all bacteria, and participate in recruiting mRNA to the 30S subunit where it is localized on the solvent side (Yusupova and Yusupov, 2014). All sequenced cyanobacteria with the exception of *Gloeobacter kilaueensis* JS1 and *Gloeobacter violaceus* PCC 7421, which lack TMs, encode two Rps1 subunits (Supplemental Figs. S6 and S7). Therefore, it is possible Rps1 subunits may play a role in determining protein localization to different subcellular locations. Rps3 is thought to form the mRNA entry tunnel along with Rps4 and Rps5 in bacteria (Ito and Chiba, 2014), and it is possible that it may play an ancillary role in anchoring a particular fraction of ribosomes to the TM. A few other proteins localized to this fraction. For example, HemA, a tRNA-Glutamyl reductase that catalyzes the first step in the heme biosynthesis pathway and uses charged tRNA-Glutamyl as a substrate, localized to the large ribosomal subunit protein fraction. In addition, Vipp1, a protein implicated in thylakoid membrane biogenesis, localized to the small ribosomal subunit protein fraction. The subcellular location and exact function of this protein in *Synechocystis* has been a matter of some controversy (Westphal et al., 2001; Hennig et al., 2015). However, localization to the ribosomal fractions is consistent with a proposed role in organizing localized protein assembly centers, as suggested by Bryan et al. (Bryan et al., 2014).

### Homologs of *Synechocystis* Thylakoid Membrane Proteins Localize to the Same Compartment in Arabidopsis (*Arabidopsis thaliana*)

In order to determine whether localization of Arabidopsis homologs of *Synechocystis* proteins have been conserved in the corresponding region of the chloroplast, proteins that have been assigned to either the TM or envelope from Arabidopsis (Ferro et al., 2010) were compared with the results obtained in this study (Supplemental Table S9). Of the TM-specific Arabidopsis homologs, six PSI, eight PSII, four cyt *b*_6_*f*, and four ATP synthase membrane bound components were identified here, in addition to nine homologs of the chloroplast NADH dehydrogenase like complex (NDH), which is known to localize to the chloroplast thylakoid membrane (Shikanai, 2016). Out of three TM-specific Arabidopsis homologs not found in these complexes, all localized to the TM in *Synechocystis*, including two hypothetical proteins (sll1390, slr1470). Therefore, 34 out of 34 TM-specific Arabidopsis homologs localized to the same membrane in *Synechocystis*. Of the 31 homologous Arabidopsis chloroplast envelope proteins, 22 were identified in *Synechocystis*, with ten in the PM and seven in the TM, while the remainder were unclassified. Of these seven, two are involved in lipid biosynthesis. In Arabidopsis, the essential pathway for thylakoid lipid biosynthesis requires export of fatty acids from the chloroplast to the endoplasmic reticulum (Xu et al., 2005). This suggests that a number of TM localized processes have been transferred to the envelope in chloroplasts during evolutionary remodeling, presumably to accommodate the requirements of organelle function in a eukaryotic cell. One protein, Sll0269, associated with the small ribosomal subunit region. Proteins homologous to TM specific proteins in Arabidopsis are nearly all exclusive to the TM in *Synechocystis*. Of the remaining 62 uncharacterized TM localized proteins in *Synechocystis*, 10 (slr1747, sll0862, sll0875, sll1071, sll1399, sll1925, slr0575, slr1591, slr1821, slr1919) have homologs in *Chlamydomonas reinhardtii* and Arabidopsis, suggesting a conserved role throughout the photosynthetic lineage (highlighted in red in Supplemental Table S10). In contrast, the Arabidopsis envelope proteins are distributed in both the PM and TM of *Synechocystis*.

## DISCUSSION

Here we detail a method for separating and analyzing the cellular components of *Synechocystis*, resulting in the most extensive proteome mapping of a cyanobacterium to date. The importance of examining the whole cell compared with fractions enriched in individual compartments is highlighted by the assignment of a large number of proteins, most lacking membrane spanning domains, to the soluble fraction in our study, which had previously been assigned to membranes in the Liberton study or earlier reports using ‘purified’ fractions (e.g. Pisareva et al., 2007). In the cells examined in this study, which were cultured under continuous moderate light and carbon replete conditions, approximately two-thirds of the proteome was detected, demonstrating the advantages of this proteomics technique compared with those previously applied to map proteins in cyanobacteria. In certain cases the technique described here allowed identification of the isoenzyme catalyzing specific biosynthetic steps under these conditions. For example, only one of the two possible Asp aminotransferases (Sll0402) was detected. The remaining proteome may not have been detected for a variety of reasons. Only proteins that were identified in both replicates were included, and, although MS is a sensitive method, some proteins may be expressed at levels too low to be detected via this approach. Other proteins may simply not be expressed under these conditions. Examples of this include proteins expressed only under microoxic conditions such as Ho2, involved in phycobiliprotein biosynthesis, and PsbA1, a subunit of PSII (Summerfield et al., 2008), and conditions of low carbon dioxide availability, such as the flavodiiron proteins Flv2 and Flv4 (Zhang et al., 2012). Of the 1,227 potential proteins not detected, 444 were hypothetical proteins and 360 were unknown. It is possible that the genes encoding these proteins may not produce functional products or be transcriptionally inactive. Regardless, the development of a robust technique for separating cellular components will facilitate proteomics of *Synechocystis* cultured under a range of environmental conditions. This technique may also be useful for analyzing the proteome of other cyanobacteria and possibly microalgae, especially because membrane separation techniques are poorly developed in unicellular photosynthetic species apart from *Synechocystis* and are not ideal due to large amounts of cellular material being lost. Other prokaryotes that have complicated internal structures, such as purple photosynthetic bacteria, or complex multilayered cell walls, for example Corynebacterineae, may also benefit from analysis via these methods.

The higher proportion of proteins detected and localized to specific regions of the cell in this study compared with published data using purified membranes further emphasizes the advantages of this method. Purification of only a subfraction of cellular components in past studies may explain this difference. The heterogeneous nature of the membranes and cytoplasm of *Synechocystis* is illustrated by the existence of subregions within the PCA plot (Fig. 5A). Particularly intriguing was the presence of possible subfractions in the PM and a region that may correspond to the OM. Although it is not possible for us to define these regions currently, due to our lack of knowledge of their composition, previous studies have suggested a heterogeneous distribution of proteins within the PM and TM (Srivastava et al., 2006; Agarwal et al., 2010; Straskova et al., 2019). As our understanding of processes within the cells increases, other regions, or subregions, may be identified. For example, as the proteins embedded within the OM become better identified and characterized we can integrate this into our model to carry out further predictions of the proteome of this region.

The complexity of cyanobacteria compared with other prokaryotes is likely to be due to the requirement to separate photosynthesis into a separate compartment, which is supported by our results. The majority of metabolic enzymes are soluble, whereas the TM and PM have specialized roles focusing primarily on energy conversion and transport, respectively (Fig. 6). Although this is obviously a successful evolutionary strategy, the presence of multiple compartments, further complicated by the presence of subregions within the membranes and possibly the cytosol, means that these organisms require a complex targeting system capable of directing proteins to the correct location. How this occurs is still poorly understood (Frain et al., 2016). Subunits of the protein translocation systems localized only to the TM, although it is possible that a small proportion are present in the PM. Intriguingly, the leader peptidase LepB1 localized specifically to the PM. Therefore, it is possible that this protein has a role in targeting proteins specifically to this membrane. Another possibility is that mRNAs migrate to specific subcellular locations (Nevo-Dinur et al., 2011; Moffitt et al., 2016) and that following translation proteins are inserted into the membrane or region in closest proximity. This is a distinct possibility given the spatial distribution of ribosomes throughout the cell. Furthermore, ribosomes on membrane-like structures connected to the TM have been observed in *Synechocystis* (van de Meene et al., 2006). Certain ribosomal subunits, such as TM localized Rps3 and cytosolic Rps1A and Rps1B, may have a role in anchoring ribosomes to different cellular regions. Our study has also provided insights into the proteomic remodeling associated with the evolution of a chloroplast from a cyanobacterium.

Although the method developed as part of this study has achieved the most extensive subcellular map of *Synechocystis* to date, the approach is not without some limitations. Although subunits of some protein complexes colocalized on the PCA plot, others may have dissociated from one another during sample preparation, and in the future it would be interesting to compare these data with those obtained using a workflow that employs protein crosslinking reagents (Liu et al., 2015; Leitner et al., 2016). Furthermore, the data visualization methods employed use a dimension reduction approach and it cannot be ruled out that the apparent resolution of some unrelated cellular substructures is lost as a result of this or by the physical subcellular separation methods employed. In the future it would be interesting to see how the map presented here compares with similar data achieved using different cell fractionation methods such as differential centrifugation and free flow electrophoresis, or other spatial approaches involving proximity tagging (Lam et al., 2015; Kim et al., 2016; Loh et al., 2016). Ultimately, our knowledge of many aspects of cyanobacterial biology is poor, with function assigned to only about 50% of genes in *Synechocystis* (http://genome.annotation.jp/cyanobase), the most highly characterized species within the phylum. Because the majority of the proteins identified in this study have no assigned function, understanding their location in the cell will aid future studies characterizing their exact role. For example, Slr0060, currently classified as an unknown protein, may be associated with PHB granules due to its proximity to PhaE, PhaC, and PhaP in our data. Of particular interest are the 10 TM localized, uncharacterized proteins that have homologs in *C. reinhardtii* and Arabidopsis, which are likely to have a conserved role in photosynthesis.

This database is the largest and most extensive list of the *Synechocystis* TM and PM proteome and is an invaluable tool to identify how proteins are targeted to each compartment and how these mechanisms could be used to insert recombinant proteins into different membrane compartments for biotechnology applications, that is, the insertion of transporters into the PM for export of biofuels and industrial compounds.

## METHODS

### Bacterial Strains, Media, and Growth Conditions

*Synechocystis* sp. PCC 6803 was routinely cultured in liquid BG11 medium with 10 mm sodium bicarbonate (Castenholz, 1988) at 30°C and grown under continuous moderate white light (50 µmol photons m^−2^ s^−1^) with vigorous air bubbling and shaking at 160 rpm. For growth of larger cultures, two 50-mL starter cultures were grown for 3 to 4 d in BG11 medium with 10 mm sodium bicarbonate to OD_750nm_ = ∼1 and used to inoculate 2 × 2 L flasks containing 1 L of BG11 medium with 10 mm sodium bicarbonate. Cultures were air bubbled and harvested at OD_750nm_ = ∼2.

### Cell Lysis and Subcellular Fractionation

Whole-cell lysate was fractionated by Suc density ultracentrifugation, as previously described (Schottkowski et al., 2009), with modifications. All steps were carried out at 4°C. Cells were harvested from 2 l cultures, by centrifugation at 5,000*g* for 10 min. The cell pellet was washed in 50 mL buffer I (5 mm Tris-HCl, pH 6.8) and centrifuged at 5,000*g* for 10 min. The resulting cell pellet was resuspended in 75 mL buffer II (10 mm Tris-HCl, 1 mm phenylmethylsulfonyl fluoride, 600 mm Suc, 5 mm EDTA, 0.2% [w/v] lysozyme, pH 6.8) and shaken at 160 rpm for 2 h at 30°C before centrifugation at 5,000*g* for 10 min. The cell pellet was washed twice with buffer III (20 mm Tris-HCl, 1 mm phenylmethylsulfonyl fluoride, 600 mm Suc, pH 6.8) and resuspended in 17.5 mL of the same buffer, to which half the volume of 425–600 µm acid-washed glass beads was added. Cells were disrupted in a Mini Bead Beater-16 (BioSpec Products) for 10 min at 3,450 oscillations/min, with 1-min intervals on ice. The cell suspension was centrifuged at 3,000*g* for 10 min to pellet unbroken cells. The supernatant was concentrated to 50% Suc by the addition of 80% Suc (w/w) in buffer II to a final volume of 10 mL. The refractive index of Suc solutions was measured to ensure correct concentrations by using a hand-held refractometer (Reichert). A discontinuous Suc gradient containing buffer II was made, consisting of 10 mL 50% (w/w) including cell lysate, 8 mL 39% (w/w), 6 mL 30% (w/w), and 6 mL 10% (w/w), and centrifuged at 125,000*g* for 17 h (SW 32 Ti Swinging Bucket Rotor, Beckman Coulter Optima l-100 XP Ultracentrifuge). Fractions 10% (I), 30% (II), 39% (III), and 50% (IV) were collected using a fraction collector (LabConco). Fraction V was diluted with 5 mm Tris-HCl buffer (pH 6.8) to a concentration of 20% (w/w) and added onto a continuous Suc gradient from 30% (w/w) to 60% (w/w) and centrifuged at 125,000*g* for 17 h. The 2.5-mL fractions were collected (1-12) using a fraction collector.

Protein precipitation was performed using a methanol-chloroform system (chilled methanol/chloroform/water, 4:1:3 [v/v/v]; Wessel and Flügge, 1984]. Protein was recovered at the interphase after vigorous vortexing for 30 s and centrifugation at 13,000*g* for 90 s at 4°C. The upper phase was discarded and the protein disc washed in 3 volumes of methanol before further centrifugation (13,000*g*, 90 s, 4°C) to pellet the protein, which was air-dried after removal of the supernatant. Protein pellets were solubilized by resuspension in 150 µL 50 mm HEPES-NaOH and 0.2% (w/v) SDS (pH 7.4), and incubated at 42°C for 15 min. Protein concentration was determined using the DC Protein Assay kit (Bio-Rad).

### SDS-PAGE and Immunoblotting

Samples from each of the fractions collected were boiled in 4 × Laemmli sample buffer for 10 min. Proteins were resolved on a 4% to 20% SDS-PAGE gel (Bio-Rad), transferred to PVDF membrane (Amersham Hybond-P, 0.45 µm; GE Healthcare), and detected with antibodies against PM (SbtA, 1/2,000; Agrisera)- and TM (CP47, 1/2,000; Agrisera)-specific proteins (Norling et al., 1998; Zhang et al., 2004) by chemiluminescence using westernBright Quantum Blotting Detection Reagent (Advansta). Visualization was carried out using a G:Box imaging system (Syngene).

### Protein Digestion and TMT 10-Plex Labeling

Suc gradient fractions 1 and 2, as well as 11 and 12, were combined, leaving 10 samples for TMT 10-plex labeling. Each sample was normalized to 100 µg of protein in 25 mm Triethylammonium bicarbonate, before being reduced, alkylated, and digested with trypsin. Each sample was made up to a total volume of 50 µL with 25 mm Triethylammonium bicarbonate. Disulphide bonds were reduced with 5 µL 200 mm tris(2-carboxyethyl)phosphine for 1 h at 55°C, followed by alkylation of Cys residues with 5 µL of 375 mm iodoacetamide for 20 min at room temperature in the dark. Protein was precipitated from the samples by addition of 6 volumes of ice-cold acetone, vortexing, and incubation at −20°C overnight. The protein pellet was recovered by centrifugation at 16,000*g* for 10 min, air-dried, and solubilized in 100 µL 100 mm HEPES (pH 8.5). Samples were digested with 2.5 µg sequencing grade trypsin (Promega) for 1 h at 37°C. A second aliquot of 2.5 µg trypsin was added to the samples, and incubated at 37°C overnight. Trypsin digests were centrifuged for 10 min at 13,000*g* to remove any insoluble material.

The 10 TMT tags were equilibrated to room temperature and resuspended in 41 µL acetonitrile before being added to each of the 10 peptide samples. Samples were placed onto a shaker for 2 h at room temperature. TMT labeling efficiency was between 93% and 95%. Unreacted TMT tags were quenched with 8 µL 5% (w/v) hydroxylamine in 100 mm HEPES (pH 8.5) for 1 h at room temperature. Ultrapure water (100 µL) was added and the samples incubated at 4°C overnight. The samples were then combined and reduced to dryness by vacuum centrifugation.

The solid-phase extraction of TMT-labeled peptides was performed according to the method previously described (Villén and Gygi, 2008), with modifications. The samples were resuspended in 1 mL of 0.4% (v/v) formic acid, and placed onto 100 mg Sep Pak tC28 solid phase extraction cartridges (Waters Corporation). Cartridges were conditioned using 1.8 mL 100% (v/v) acetonitrile, followed by 50% (v/v) acetonitrile and 0.5% (v/v) acetic acid, and equilibrated with 1.8 mL 0.1% (v/v) formic acid. The peptides were de-salted after loading in 1.8 mL 0.1% (v/v) formic acid, re-equilibrated with 500 µL 0.5% (v/v) acetic acid. Samples were eluted with 0.5 mL 75% (v/v) methanol with 0.5% (v/v) acetic acid, followed by 75% (v/v) acetonitrile with 0.5% (v/v) acetic acid, and reduced to dryness by vacuum centrifugation before resuspension in 0.1-mL 20 mm ammonium formate (pH 10), 4% (v/v) acetonitrile, for high pH reversed-phase liquid chromatography.

### Sample Fractionation

Peptides were loaded onto an Acquity bridged ethyl hybrid C18 UPLC column (Waters; 2.1 mm i.d. × 150 mm, 1.7 µm particle size) and profiled with a linear gradient of 5% to 75% acetonitrile + 20 mm ammonium formate (pH 10) over 50 min, at a flow rate of 50 µL/min. Chromatographic performance was monitored by sampling eluate with a diode array detector (Acquity UPLC, Waters) scanning between wavelengths of 200 and 400 nm. Forty-four fractions were collected from 11 min onward in 1-min intervals. Fractions 1–8 were pooled together, and the rest were pooled pairwise, with fraction 9 pooled with fraction 26, 10 with 27, and so on to yield 19 samples for mass spectrometry analysis.

### Mass Spectrometry

All LC-MS/MS experiments were performed using a Dionex Ultimate 3000 RSLC nanoUPLC (Thermo Fisher Scientific) system and a Lumos Fusion Orbitrap mass spectrometer (Thermo Fisher Scientific) using synchronous precursor selection-MS. Each of the fractionated samples was resuspended in 35 µL 0.1% (v/v) formic acid and between 1 and 5 µL of these was applied to LC-MS/MS analysis using an Orbitrap Fusion Lumos coupled with a Proxeon EASY-nLC 1000 (Thermo Fisher Scientific). Separation of peptides was performed by reverse-phase chromatography at a flow rate of 300 nL/minute and a Thermo Scientific reverse-phase nano Easy-spray column (Thermo Scientific PepMap C18, 2 μm particle size, 100A pore size, 75 μm i.d. × 50 cm length). Peptides were loaded onto a precolumn (Thermo Scientific PepMap 100 C18, 5 μm particle size, 100A pore size, 300 μm i.d. × 5 mm length) from the Ultimate 3000 autosampler with 0.1% (v/v) formic acid for 3 min at a flow rate of 10 μL/min. After this period, the column valve was switched to allow elution of peptides from the precolumn onto the analytical column. Solvent A was water + 0.1% (v/v) formic acid, and solvent B was 80% acetonitrile, 20% water + 0.1% (v/v) formic acid. The linear gradient used was 4-140 B in 100 min (the total run time including column washing and re-equilibration was 120 min).

An electrospray voltage of 2.1 kV was applied to the eluent via the EASY-Spray column electrode. The following workflow in the Method Editor was used: MS OT (Detector type: Orbitrap, Resolution: 120000, Mass range: Normal, Use Quadrupole Isolation [Yes], Scan Range: 380–1500, RF Lens [%]: 30, AGC Target: 4e5, Max Inject Time: 50 ms, Microscans: 1, Data Type: Profile, Polarity: Positive) - Monoisotopic Precursor Selection (MIPS; Monoisotopic Peak Determination: Peptide, Relax restrictions when too few precursors are found: Yes) - Charge State (Include charge state(s): 2-7) - Dynamic Exclusion [Exclude after n times: 1, Exclusion duration (s): 70, Mass Tolerance; ppm, Low: 10, High: 10, Exclude Isotopes: Yes, Perform dependent scan on single charge state per precursor only: Yes] - Intensity Threshold (5.0e3) - Decisions (Data dependent mode: Top Speed, Number of Scan Event Types: 1, Scan Event Type 1: No Condition) - ddMS2 IT CID (MSn Level: 2, Isolation Mode: Quadrupole, Isolation Window [*m/z*]: 0.7, Activation Type: CID), CID Collision Energy (%): 35, Activation Q: 0.25, Detector Type: Ion Trap, Scan Range Mode: Auto, *m/z*: Normal, Ion Trap Scan Rate: Turbo, AGC Target; 1.0e4, Max Inject Time (ms): 50, Microscans: 1, Data Type: Centroid) - Precursor Selection Range (Mass Range: 400–1200) - Precursor Ion Exclusion (Exclusion mass width: *m/z*, Low: 18, High: 5) - Isobaric Tag Loss Exclusion (Reagent: TMT) - Decisions (Precursor Priority: Most Intense, Scan event type 1: No Condition) - ddMS3 OT HCD (Synchronous Precursor Selection: Yes, Number of Precursors: 10, MS Isolation Window: 0.7, Activation Type: HCD, HCD Collision Energy [%]: 65, Detector Type: Orbitrap, Scan Range Mode: Define *m/z* range, Orbitrap Resolution: 60000, Scan Range [*m/z*]: 100-500, AGC Target: 1.0e5, Max Inject Time [ms]: 120, Microscans: 1, Data Type: Profile). Total run time was 120 min.

### Data Processing

Raw data files were processed using Proteome Discoverer (v1.4.1.14, Thermo Fisher Scientific), interfaced with Mascot server (v.2.3.02, Matrix Science). Mascot searches were performed against the CyanoBase database, with carbamidomethylation of Cys, and TMT 10-plex modification of Lys and peptide N termini set as modifications. Precursor and fragment ion tolerances of ± 20 p.p.m and ± 0.1 D were applied. Up to 2 missed tryptic cleavages were permitted. Proteins were reported with a false discovery rate of 0.5%.

TMT 10-plex quantification was also performed via Proteome Discoverer by calculating the sum of centroided ions within a ± 2 mmu window around the expected *m/z* for each of the 10 TMT reporter ions. For protein-level reporting, protein grouping was enabled, and values were calculated from the median of all quantifiable peptide spectral matches (PSMs) for each group. TMT values were then reported as a ratio to the sum of reporters in each spectrum.

### Machine Learning, Multivariate Analysis, and Visualization of Data

The Bioconductor (Gentleman et al., 2004) packages MSnbase (Gatto and Lilley, 2012) and pRoloc (Gatto et al., 2014) for the R statistical programming language (R Core Team, 2013) were used for handling of the quantitative proteomics data and the protein-localization prediction. pRolocGUI (Gatto et al., 2014) was used for interactive visualization of the data. Protein markers for the plasma membrane, thylakoid membrane, cytosol, and small and large ribosomal subunits were curated from a literature review (Supplemental Table S8). An SVM classifier was used on the combined dataset, with a radial basis function kernel, using class specific weights for classification of unassigned proteins to one of the five defined subcellular niches, TM, PM, soluble, small ribosomal subunit, or large ribosomal subunit. The weights used in classification were set to be inversely proportional to the subcellular class frequencies to account for class imbalance. Algorithmic performance of the SVM on the dataset was estimated (as described in Trotter et al., 2010). Scoring thresholds were calculated per subcellular niche and were set based on concordance with existing subcellular knowledge annotation to attain a 7.5% false discovery rate. Unassigned proteins were then classified to one of the five compartments according to the SVM prediction if greater than the calculated class threshold.

All protein level datasets are available in the R Bioconductor pRolocdata package (https://bioconductor.org/packages/pRolocdata version 1.19.2) and can be interactively explored using the pRolocGUI package (https://bioconductor.org/packages/pRolocGUI) or using the standalone online interactive app (https://lgatto.shinyapps.io/synechocystis/).

The mass spectrometry data have been deposited to the ProteomeXchange Consortium (http://proteomecentral.proteomexchange.org) via the PRIDE (Perez-Riverol et al., 2019) partner repository with the dataset identifier PXD014662.

### Accession Numbers

Gene/protein names, products, and accession numbers of all genes/proteins identified in this study are listed in Supplemental Table S3.

### Supplemental Data

The following supplemental materials are available.

**Supplemental Figure S1.** Growth of *Synechocystis* under continuous moderate light (60 µmol photons m^−2^ s^−1^) with air-bubbling at 30°C.
**Supplemental Figure S2.** Partial fractionation of *Synechocystis* by Suc density ultracentrifugation. Lysed cells were fractionated based on the method by Schottkowski et al. (2009) with modifications.
**Supplemental Figure S3.** Comparison of assignment of proteins from this study with the Liberton et al. (2016) data set between: A. Those found in the membranes in both studies and B. Those found in the soluble fraction in this study.
**Supplemental Figure S4.** Carotenoid biosynthesis in *Synechocystis*.
**Supplemental Figure S5.** Distribution of carboxysome subunits and internal proteins in the PCA plot.
**Supplemental Figure S6.** Alignment of Rps1A subunits from sequenced cyanobacterial species.
**Supplemental Figure S7.** Alignment of Rps1B subunits from sequenced cyanobacterial species.
**Supplemental Figure S8.** Comparison of the TM and PM proteomes in terms of their functional categories.
**Supplemental Table S1.** Large-scale proteomic studies of *Synechocystis*.
**Supplemental Table S2.** TMT quantitation data for two LOPIT replicate experiments and length, weight and pI of proteins identified.
**Supplemental Table S3.** Proteins identified in both replicates, the predicted localisations of proteins in *Synechocystis* by machine learning, using marker proteins as a training set.
**Supplemental Table S4.** Proteins identified in this study and the one performed by Spät et al. (2018).
**Supplemental Table S5.** Proteins identified in this study but not the one performed by Spät et al. (2018).
**Supplemental Table S6.** Proteins not identified in this study but identified in the one performed by Spät et al. (2018).
**Supplemental Table S7.** Proteins not identified in this study or in the one performed by Spät et al. (2018).
**Supplemental Table S8.** Marker proteins used to identify subcellular regions.
**Supplemental Table S9.** Comparison of the localization of Arabidopsis chloroplast envelope and thylakoid membrane proteins with homologs in *Synechocystis*.
**Supplemental Table S10.** BLAST analysis of uncharacterized *Synechocystis* TM localized proteins
